# Integrated Analysis of Clinical Outcome of Mesenchymal Stem Cell-related Genes in Pan-cancer

**DOI:** 10.2174/0113892029291247240422060811

**Published:** 2024-04-26

**Authors:** Mingzhe Jiang, Dantong Zhu, Dong Zhao, Yongye Liu, Jia Li, Zhendong Zheng

**Affiliations:** 1 Department of Oncology, General Hospital of Northern Theater Command, Shenyang, China

**Keywords:** Mesenchymal stem cells, immunotherapy, pan-cancer, neural networks, clinical prognosis, cox regression analysis

## Abstract

**Background:**

Although the application of mesenchymal stem cells (MSCs) in engineered medicine, such as tissue regeneration, is well known, new evidence is emerging that shows that MSCs can also promote cancer progression, metastasis, and drug resistance. However, no large-scale cohort analysis of MSCs has been conducted to reveal their impact on the prognosis of cancer patients.

**Objectives:**

We propose the MSC score as a novel surrogate for poor prognosis in pan-cancer.

**Methods:**

We used single sample gene set enrichment analysis to quantify MSC-related genes into a signature score and identify the signature score as a potential independent prognostic marker for cancer using multivariate Cox regression analysis. TIDE algorithm and neural network were utilized to assess the predictive accuracy of MSC-related genes for immunotherapy.

**Results:**

MSC-related gene expression significantly differed between normal and tumor samples across the 33 cancer types. Cox regression analysis suggested the MSC score as an independent prognostic marker for kidney renal papillary cell carcinoma, mesothelioma, glioma, and stomach adenocarcinoma. The abundance of fibroblasts was also more representative of the MSC score than the stromal score. Our findings supported the combined use of the TIDE algorithm and neural network to predict the accuracy of MSC-related genes for immunotherapy.

**Conclusion:**

We comprehensively characterized the transcriptome, genome, and epigenetics of MSCs in pan-cancer and revealed the crosstalk of MSCs in the tumor microenvironment, especially with cancer-related fibroblasts. It is suggested that this may be one of the key sources of resistance to cancer immunotherapy.

## 1 INTRODUCTION

Mesenchymal stem cells (MSCs) are multipotent progenitor cells that can differentiate into many cell types [1, 2], have a significantly higher differentiation potential [3, 4], and play reparative roles in several organs, such as the lungs [5], liver [6], brain [7], and heart [8]. Numerous studies have focused on the *in vivo* properties of MSCs in tissue regeneration; however, few have determined their roles in primary tumor tissues and at metastatic sites. Accumulating evidence shows that MSCs are capable of migrating to the tumor microenvironment (TME) [9-12].

There is a direct regulatory relationship between MSCs and cancer cells. Cancer cells exhibit a principal feature of interacting with the surrounding matrix, with these interactions resulting in an ‘activation state’ that leads to elevations in proinflammatory cytokine levels and the production of growth factors [13], which promote the recruitment of responsive cell types [14, 15]. During the tumor inflammatory stage, tumor-associated MSCs (TA-MSCs) perform a similar function to that exhibited during wound healing. TA-MSCs have been cultured within the inflammatory TME and are generated from healthy MSCs at the site of carcinogenesis [16, 17]. Furthermore, MSCs can influence the TME through indirect processes. MSCs derived from resident and distant tissues are considered tumor-associated fibroblast (TAF) precursors. Kidd *et al.* [18] quantitatively assessed the origin of TAFs in the TME *in vivo* in a syngeneic mouse model of ovarian cancer, finding that ~40% of tumor stromal cells were generated from the bone marrow. Additionally, they reported that most TAFs positive for fibroblast-specific protein and fibroblast-activating protein were derived from bone marrow-MSCs, whereas α-SMA+TAF and perivascular stromal cells (pericytes) were mainly derived from the adipose tissues near the tumor [18].

Therefore, in this study, we comprehensively characterized the genomic variation and expression profiles of pan- cancer MSCs and patient prognosis. Moreover, we applied computational modeling to explore the possible efficacy of immunotherapy based on the expression profiles of MSCs. The findings offer insights into the possible targeting of MSCs for the treatment of cancer patients, as well as the benefits of employing machine learning to determine potential clinical applications for immunotherapy.

## 2 MATERIALS AND METHODS

### 2.1 Datasets and Source Code

mRNA-expression and masked somatic mutation data were downloaded from The Cancer Genome Atlas (TCGA) and Genomic Data Commons portal (https://portal.gdc.cancer.gov/). They include data related to bladder urothelial carcinoma (BLCA), adrenocortical carcinoma (ACC), cholangiocarcinoma (CHOL), breast invasive carcinoma, colon adenocarcinoma (COAD), cervical and endocervical cancer (CESC), esophageal carcinoma, lymphoid neoplasm diffuse large B-cell lymphoma (DLBC), glioblastoma multiforme (GBM), acute myeloid leukemia (LAML), glioma (LGG), kidney chromophobe (KICH), liver hepatocellular carcinoma (LIHC), kidney renal clear cell carcinoma (KIRC), head and neck squamous cell carcinoma, lung adenocarcinoma, ovarian serous cystadenocarcinoma (OV), pancreatic adenocarcinoma (PAAD), kidney renal papillary cell carcinoma (KIRP), mesothelioma (MESO), pheochromocytoma and paraganglioma, rectum adenocarcinoma (READ), skin cutaneous melanoma (SKCM), lung squamous cell carcinoma (LUSC), prostatic adenocarcinoma (PRAD), thyroid carcinoma (THCA), sarcoma, uveal melanoma (UVM), stomach adenocarcinoma (STAD), testicular germ cell tumors (TGCTs), thymoma (THYM), uterine carcinosarcoma (UCS), and uterine corpus endometrial carcinoma.

Copy number variation data were derived from cBioPortal for Cancer Genomics (https://www.cbioportal.org/). Data on the methylation levels of the promoter regions of MSC-related genes in pan-cancer (only for those with both tumors and normal samples) were obtained from the UALCAN database (http://ualcan.path.uab.edu/). Clinical data were downloaded from the Xena Browser (https://xenabrowser.net/datapages/).

Estimation of STromal and Immune cells in MAlignant Tumors using Expression data [19] was used to assess the TME composition, with the specific immune cells and matrix components of the TME derived from CIBERSORT [20] and TCIA [21], respectively.

TIDE [22] was used to evaluate the response to immune-checkpoint therapy and calculate the immune-escape scores. Connectivity Map (CMap) [23] was used to determine the compounds and inhibitors capable of targeting MSC-related genes. Using the Gene Expression Omnibus (https://www.ncbi.nlm.nih.gov/geo/), we obtained the microarray datasets, including the gene-expression profiles for Cox analysis validation for GSE107850 and Chinese Glioma Genome Atlas (http://www.cgga.org.cn/) of LGG, GSE29354 of MESO, and GSE13181, GSE15459, GSE62254, and GSE84437 of STAD. The seven validation datasets of immune-checkpoint therapy were based on IMvigor210CoreBiologies [24], GSE78220, GSE135222, GSE165252, GSE79671, PRJEB25780, and GSE176307 from urothelial carcinoma, melanoma, non-small cell lung cancer, esophageal adenocarcinoma, glioblastoma, and metastatic gastric cancer to metastatic urothelial cancer, respectively (Supplementary Tables **1** and **2**).

### 2.2 Differential mRNA-expression Analysis of MSC- related Genes

To explore the differential expression of 59 MSC-related genes [25], we used normal human tissue samples from Genotype–Tissue Expression (GTEx, https://commonfund.nih.gov/GTEx/) as controls, given that there are no normal mRNA samples for ACC, DLBC, LAML, LGG, OV, SKCM, OV, SKCM, TGCTs, and UCS in TCGA, and there is only one normal sample for SKCM. The relevant data were restored to the same type as the FPKM data of TCGA for subsequent processing and analysis. The fold-change was set to ≥2, and a *P* < 0.05 was considered statistically significant.

### 2.3 Calculation of the MSC Score

The MSC score was calculated based on the results of single-sample gene set enrichment analysis (GSEA) using the MSC-related gene set to quantify the expression levels of these genes for each cancer type [26]. We estimated the MSC score between the tumor tissue and normal tissue samples from TCGA datasets with supplemental normal samples in GTEx. We grouped the high and low MSC score groups according to the median MSC score for each cancer type. Distribution of enrichment scores of MSCs in a human anatomy diagram with gganatogram package (https://github.com/neuroconductor/gganatogram).

### 2.4 Survival Analysis

We compared the progression-free interval (PFI), disease-free interval, disease-specific survival (DSS), and overall survival (OS) of patients with cancer stratified by the median MSC score for each type of cancer. Kaplan–Meier curves were used to compare the differences in the survival of patients, and *P*-values were calculated using log-rank tests, with *P* < 0.05 considered statistically significant.

### 2.5 Immune Activity, Tumor Mutation Burden, and Fibroblast-related Signature Enrichment in Tumor Evaluation

To assess the TME, we used Estimation of STromal and Immune cells in MAlignant Tumors using Expression data, which determines the immune and stromal scores for each tumor sample. To quantify the abundance of fibroblasts in the TME, we used three methods: Xcell [27], MCPcounter [28], and EPIC [29]. The tumor mutation burden was estimated for each tumor sample as the total number of somatic mutations detected in the tumor.

### 2.6 Neural Network Construction

To evaluate the immunotherapeutic response, we constructed a neural network using PyTorch based on the signatures of MSC-related genes in Python (v3.8.8) [30]. The stochastic gradient descent method was utilized to optimize the model using a learning rate of 0.001. Five layers were built using different input and output numbers. Subsequently, batch normalization was performed on each layer.

The dropout function (dropout rate, 0.2) was utilized in the training phase. The ReLU function was applied as an activation function, and a logistic sigmoid function was utilized for the output layer (Supplementary Fig. **S1**).

### 2.7 GSEA

Based on the median MSC score, each tumor sample was classified into high or low score groups for determining the pathways associated with the MSC score, and GSEA was performed [31].

### 2.8 Statistical Analyses

The Wilcoxon test was used to compare differences in the MSC score between cancer tissue and adjacent or normal tissue, wild-type and mutant genes, the tumor mutation burden and microsatellite-instability of the high and low MSC groups, and between the response and non-response groups. Spearman’s correlation analysis was used to analyze the correlation between MSC score and matrix and immune components. All analyses were performed using R software (v4.0.3; https://www.r-project.org/).

## 3 RESULTS

### 3.1 MSC Score According to GSEA and Differential Expression of MSC-related Genes in Pan-cancer

**Fig. (1):**
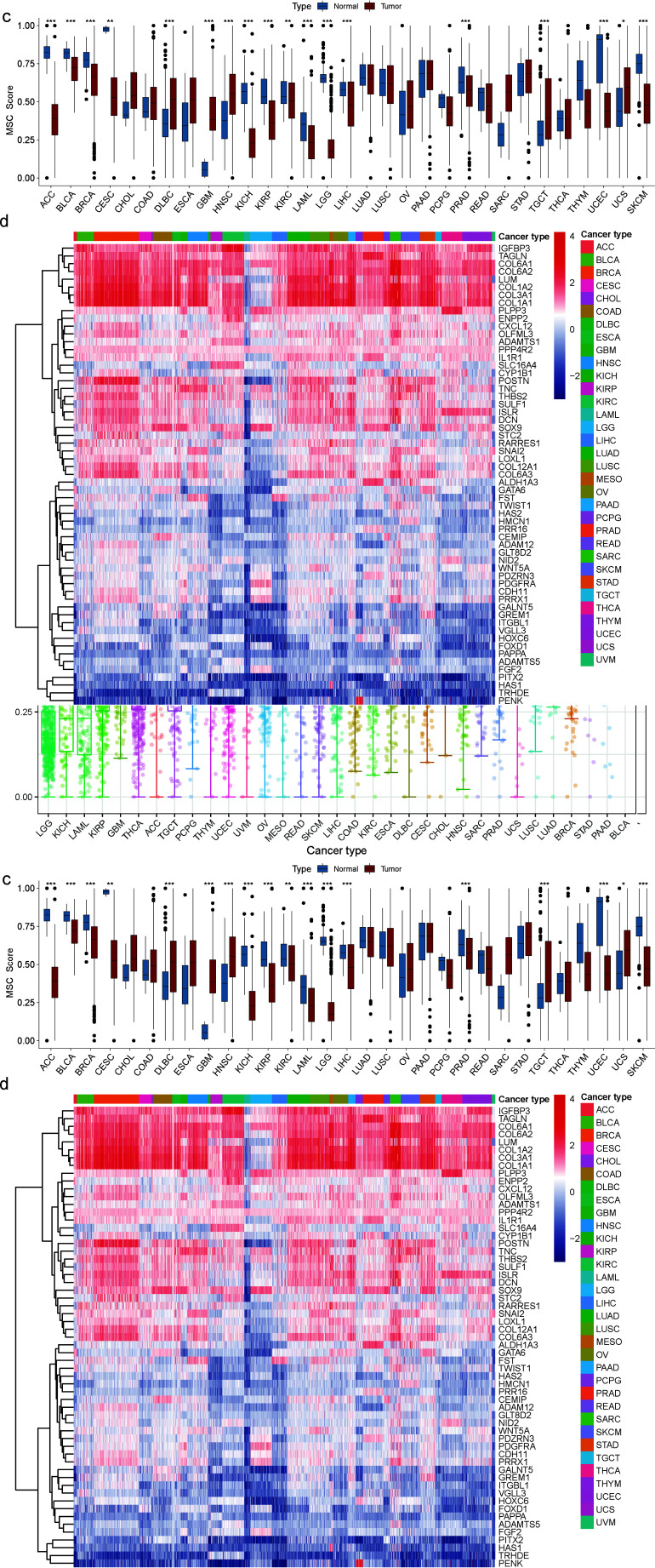
MSC score and differential MSC-related gene expression in pan-cancer of TCGA and GTEx. (**a**) Distribution of enrichment scores of MSCs in a human anatomy diagram with gganatogram package (https://github.com/neuroconductor/gganatogram) of TCGA and GTEx. (**b**) MSC scores of all samples grouped according to the 33 cancer types in TCGA dataset. *P < 0.05, **P < 0.01, ***P < 0.001. (**c**) Comparison of the enrichment score of MSCs in normal and tumor tissues. For cancer types that lack normal samples in the TCGA dataset, we used the GTEx dataset. (**d**) Expression of the 59 MSC-related genes in the 33 cancer types based on TCGA dataset.

We used the single-sample GSEA algorithm [26] to quantify the enrichment MSC score and determine the abundance of MSCs across different cancer species. First, we characterized TCGA-based mRNA-expression profiles of 33 cancer samples, ranked from low to high, based on their differences in MSC enrichment scores (hereafter referred to as the MSC score). LGG had the lowest MSC score, whereas BLCA had the highest MSC score, with the median variation ranging from 0.1736 to 0.7223 (Fig. **1a** and **b**). Second, we verified the difference in MSC scores between normal and tumor tissues, as ACC, DLBC, LAML, LGG, OV, SKCM in TCGA (SKCM in TCGA had only one normal sample), TGCTs, and UCS lacked normal samples. Thus, normal human samples corresponding to GTEx were used to fill the gap. Eighteen tumors had statistically significant MSC scores. The MSC scores of ACC, BLCA, breast invasive carcinoma, CESCs, KICH, KIRP, KIRC, LAML, LIHC, LGG, PRAD, SKCM, and uterine corpus endometrial carcinoma were downregulated in cancer tissues relative to those in normal tissues, whereas those of GBM, head and neck squamous cell carcinoma, DLBC, TGCTs, and UCS were upregulated. Among the 59 genes in the MSC gene set, the expression levels of *IGFBP3, TAGLN, COL6A1, COL6A2, LUM, COL1A2, COL3A1*, and *COL1A1* were significantly higher, whereas those of *PITX2, HAS1, TRHDE*, and *PENK* were significantly lower than those of other MSC-related genes in the 33 cancer types (Fig. **1c** and **d**).

### 3.2 Clinical Relevance of the MSC Scores

**Fig. (3):**
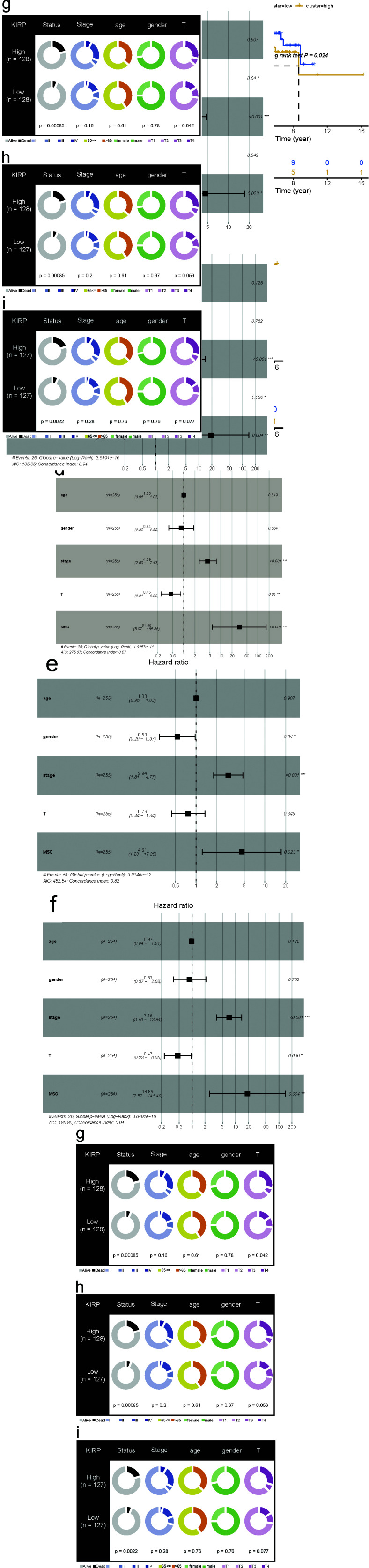
Independent prognostic factor analysis for KIRP. (**a**) OS, (**b**) PFI, and (**c**) DSS between the high and low MSC score groups. Multivariate regression analysis for MSC score and clinical characteristics in terms of (**d**) OS, (**e**) PFI, and (**f**) DSS. Fisher’s exact test between MSC score and clinical characteristics in terms of (**g**) OS, (**h**) PFI, and (**i**) DSS.

To explore the relevance of the MSC score in cancer prognosis, we first performed a univariate Cox regression analysis with pan-cancer as a predictor of OS, PFS, DSS, and disease-free interval, which were the dependent outcomes. The clinical relevance of MSC-related genes was examined across 33 cancer types, with the results shown in Fig. (**2**). Additionally, we performed Cox regression analysis to examine MSC scores as predictors of these outcomes (Supplementary Fig. **S2**). BLCA, KICH, KIRP, LGG, lung adenocarcinoma, MESO, PAAD, and STAD showed statistical significance in terms of OS; BLCA, DLBC, GBM, KICH, KIRP, KIRC, LGG, and PAAD showed statistical significance in terms of PFI; BLCA, GBM, KIRP, KIRC, LGG, MESO, PAAD, and STAD showed statistical significance in terms of DSS; and CESCs, KIRP, LIHC, PAAD, and UCSC showed statistical significance in terms of the disease-free interval. KIRP and LGG had prognostic significance in terms of OS, PFI, and DSS; MESO had prognostic significance in terms of OS and DSS; and STAD had prognostic significance in terms of OS. After combining the corresponding clinical indications, multivariate Cox regression analysis revealed the MSC scores of KIRP [TCGA-OS, hazard ratio (HR) = 31.45, 95% confidence interval (CI) = 5.97–165.55, *P* < 0.0001; TCGA-PFI, HR = 4.61, 95% CI = 1.23–17.28, *P* = 0.023; TCGA-DSS, HR = 18.86, 95% CI = 2.52–141.40, *P* = 0.004) (Fig. **3**), LGG (TCGA-OS, HR = 5.29, 95% CI = 1.06–26.38, *P* = 0.042; GSE107850-OS, HR = 5.25, 95% CI = 1.21–22.69, *P* = 0.0^27^] (Supplementary Fig. **S3**), MESO (TCGA-OS, HR = 9.41, 95% CI = 3.11–28.50, *P* < 0.0001; GSE29354-OS, HR = 4.70, 95% CI = 1.13–19.20, *P* = 0.033) (Supplementary Fig. **S4**), and STAD (TCGA-OS, HR = 22.50, 95% CI = 1.42–356.4, *P* = 0.027; GSE84437-OS, HR = 6.7, 95% CI = 1.34–33.1, *P* = 0.02; GSE15459-OS, HR = 51.23, 95% CI = 5.35–490.5, *P* < 0.0001; GSE13861-OS, HR = 181.82, 95% CI = 3.37–9806.6, *P* = 0.011; GSE13861-RFS, HR = 102.30, 95% CI = 2.191–4777.4, *P* = 0.018; GSE62254-OS, HR = 13.1, 95% CI = 2.04–84.1, *P* = 0.007; GSE62254-RFS, HR = 9.25, 95% CI = 1.26–67.8, *P* = 0.029) (Supplementary Figs. **S5** and **S6**) as potential independent prognostic factors.

### 3.3 Association between MSC Score and Cancer Pathways

We then performed functional enrichment analysis of 59 MSC-related genes using Kyoto Encyclopedia of Genes and Genomes to identify associations between the MSC score and its genes and pathways. With respect to biological processes, the MSC-related genes were mainly involved in extracellular matrix formation and changes in histological morphology. For the cellular components, MSC-related genes were associated with cytoskeleton formation, and for molecular functions, the MSC-related genes were associated with inter-cellular adhesion and growth factors, integrin, and heparin.

**Fig. (4):**
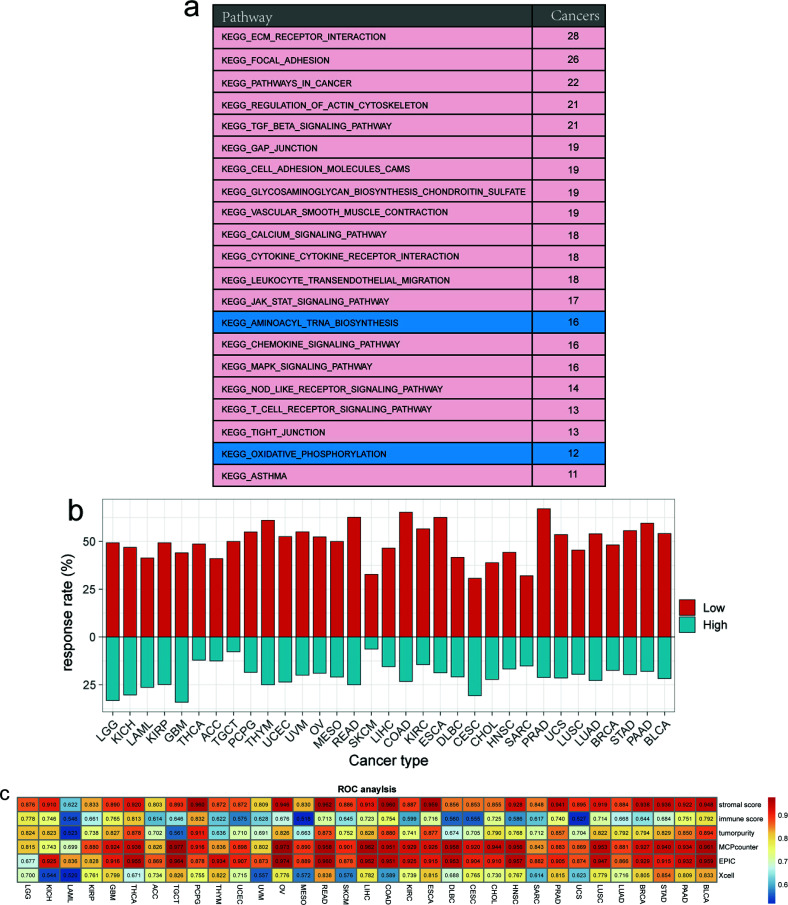
The MSC score is more predictive for stromal signatures in 33 cancer types of TCGA. (**a**) Enriched Kyoto Encyclopedia of Genes and Genomes pathways in 33 cancer types stratified according to high and low MSC scores. (**b**) Differences in the response rates predicted using TIDE between the high and low MSC score groups in pan-cancer. (**c**) Comparison of the predictive abilities of the MSC score, stromal score, immune score, and tumor purity with MCPcounter, EPIC, and Xcell for fibroblasts using ROC curve analysis.

Additionally, Kyoto Encyclopedia of Genes and Genomes analysis revealed the complex and extensive involvement of MSC-related genes in various regulatory pathways, including extracellular matrix–receptor interaction, protein digestion and absorption, focal adhesion, the PI3K–Akt signaling pathway, proteoglycans in cancer, human papillomavirus infection, the TGF-β signaling pathway, amoebiasis, and age-related signaling pathways. We classified the MSC score of each cancer type into high and low groups according to the median value, after which GSEA was used to determine the significance in >10 tumors. The high MSC score group was enriched in extracellular matrix–receptor interaction, focal adhesion, cell adhesion molecules, pathways in cancer, regulation of actin cytoskeleton, the TGF-β signaling pathway, vascular smooth muscle contraction, the calcium signaling pathway, cytokine–cytokine receptor interaction, leucocyte transendothelial migration, gap junction, glycosaminoglycan biosynthesis chondroitin sulfate, the JAK–STAT signaling pathway, the MAPK signaling pathway, the NOD-like receptor signaling pathway, the T cell receptor signaling pathway, the chemokine signaling pathway, tight junctions, and asthma pathways. By contrast, the low MSC score group was enriched in aminoacyl-tRNA biosynthesis and oxidative phosphorylation pathways (Fig. **4a**).

To reveal the potential tumor-escape mechanism of MSCs, we employed the TIDE algorithm to assess the responsiveness of each sample to immune-checkpoint drugs in TCGA pan-cancer data. First, in all 33 cancers, patients with a low MSC score had higher response rates to immune-checkpoint predictions than patients with a high MSC score. Among them, the response rate predicted for PRAD reached a maximum of 67.07%, followed by >60% for COAD, READ, esophageal carcinoma, and THYM. Moreover, the response rates were 32.77%, 32.06%, and 30.72% for SKCM, sarcoma, and CESCs, respectively. In samples with high MSC scores, the highest response rate for immune-checkpoint prediction was for GBM, followed by LGG. The response rates for CESCs and KICH exceeded 30%, whereas the lowest response rate was for SKCM (6.36%) (Fig. **4b**).

### 3.4 Fibroblast Abundance was More Representative of the MSC Score than the Stromal Score

According to a previous study performing quantitative analysis on data from animal models, at least 40% of TAFs in the TME were derived from MSCs [18]. We performed receiver operating characteristic (ROC) curve analysis to determine whether the MSC score can predict tumor immunity by predicting the stromal score, immune score, tumor purity, and fibroblast abundance. We simultaneously used three modalities (EPIC, Xcell, and MPcounter) [27-30] to quantify fibroblast abundance in each tumor sample. The MSC score showed a high potential for predicting the stromal score and was an indicator of fibroblast abundance in all 33 cancer types. By contrast, the MSC score showed better predictive power for fibroblast abundance along with MPcounter and EPIC relative to Xcell. The effect of the MSC score on the predictive ability of the immune score and tumor purity was moderate (Fig. **4c**). These results confirmed that MSCs might be a primary source of TAFs.

**Fig. (5):**
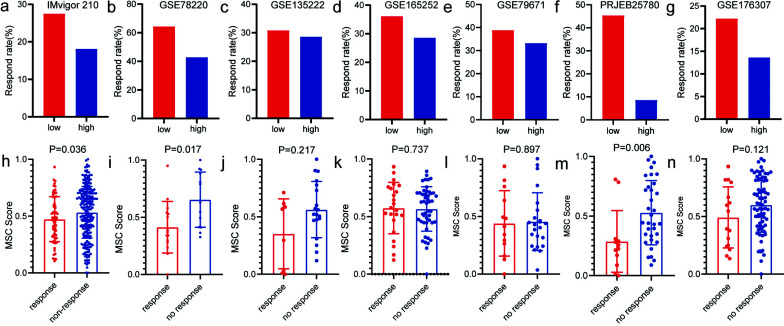
Validation of MSC scores based on predicted and real cohorts treated with immune-checkpoint inhibitors. (**a-g**) Differences in the response rates between the high and low MSC score groups in seven cohorts treated with immune-checkpoint inhibitors: IMvigor210, GSE78220, GSE135222, GSE165252, GSE79671, PRJEB25780, and GSE176307. (**h-n**) Differences in the response rates corresponding to differences in MSC scores between responder and non-responder groups: IMvigor210, GSE78220, GSE135222, GSE165252, GSE79671, PRJEB25780, and GSE176307 cohorts.

We then evaluated seven cohorts of different cancer types treated with PD-1 or PD-L1 immune-checkpoint antagonists, and the MSC scores of tumor samples from each cohort were quantified and patients were divided into the high and low MSC score groups according to a median cut-off value. Results were consistent across all cohorts: patients with tumors with low MSC scores were more responsive to PD-1 or PD-L1 immune-checkpoint antagonists than those with high MSC scores (IMvigor210, 27.52% [low] *vs*. 18.12% [high]; GSE78220, 64.28% [low] *vs*. 42.86% [high]; GSE135222, 30.77% [low] *vs*. 28.57% [high]; GSE165252, 36.11% [low] *vs*. 28.57% [high]; GSE79671; 38.89% [low] *vs*. 33.33% [high]; PRJEB25780, 45.45% [low] *vs*. 8.70% [high]; GSE176307, 22.22% [low] *vs*. 13.64% [high]). Additionally, the MSC scores of responders were generally lower than those of non-responders (Fig. **5**). These results are consistent with those derived using the TIDE algorithm to predict the effect of immunotherapy.

### 3.5 CMap Analysis Identifies Potential Compounds/inhibitors for Cancer Treatment

We used the CMap database of small-molecule drugs to identify potential therapies targeting specific differences in gene expression. The goal was to develop a new approach to improve the treatment and prognosis of most cancer types. Differential expression analysis between cancerous and para-cancerous groups across 31 cancer types (*i.e*., all except for UVM and MESO) was performed using the limma package, and genes with a *P* < 0.05 were screened as differentially expressed genes. Based on CMap data processing, 96 compounds were enriched in at least five cancer types (Supplementary Fig. **S7A**) and 19 in at least 10 cancer types (Supplementary Fig. **S7B**). The 19 compounds included 2-aminobenzenesulfonamide, deferoxamine, doxazosin, lisuride, mercaptopurine, mestranol, pheniramine, 3-acetamidocoumarin, 5182598, bumetanide, nabumetone, propylthiouracil, pyrvinium, tanespimycin, cephaeline, alvesspimycin, STOCK1N-35874, ceforanide, and hydroquinine. We used the PubChem platform (https://pubchem.ncbi.nlm.nih.gov/) to locate the three-dimensional chemical structures of 12 compounds (Supplementary Fig. **S7C**). In theory, these 19 drugs might be candidates for improving the treatment effect of most cancer types.

### 3.6 Neural Network-based Model to Identify Immunotherapy Outcomes

**Fig. (6):**
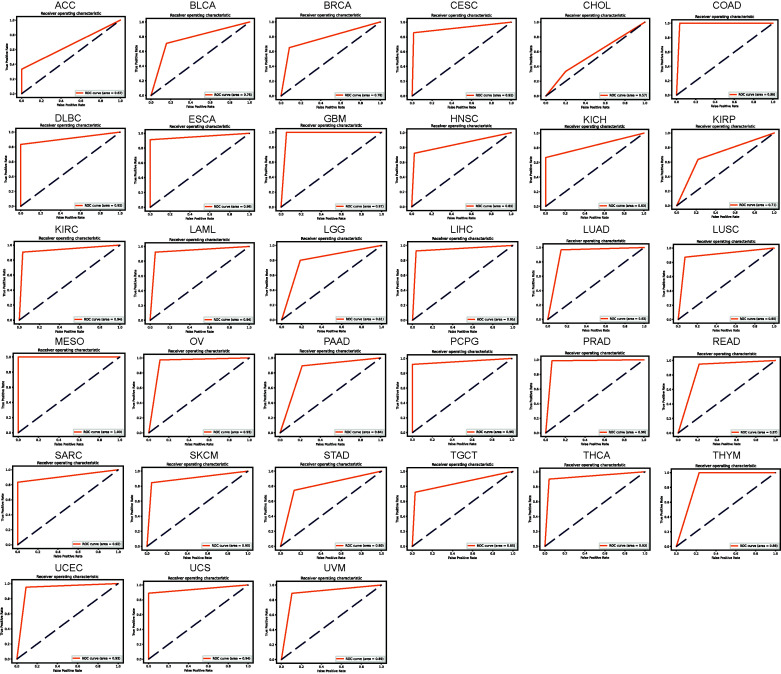
Combined use of the TIDE algorithm and a neural network to predict the accuracy of MSC-related genes for predicting immunotherapy efficacy in pan-cancer of TCGA. The area under the ROC curves was determined for predicting the clinical benefits of immunotherapy using the TIDE algorithm and a neural network.

We established a neural network-based framework in order to explore which MSC-related genes had the potential to determine which patients would benefit from immunotherapy. The neural network schematic is shown in Supplementary Fig. **S1**. The early TCGA pan-cancer dataset was divided into training and test datasets, a neural network was constructed using MSC-related gene signatures from the training dataset, and the neural network accuracy was evaluated using the test dataset. The loss value of the model decreased in the test set as the number of training epochs increased (Supplementary Fig. **S8**). The confusion matrix showed that no samples were misidentified in the COAD, GBM, MESO, and THYM test sets, and that only one sample in the DLBC, LAML, OV, PRAD, READ, UCS, and UVM test sets was misidentified. The number of samples in which other tumors were misidentified ranged from 2 to 42 (Supplementary Fig. **S9**). The ROC curves for COAD, GBM, MESO, and THYM showed high accuracies, with areas under the curve reaching 0.98, 0.97, 1.00, and 0.88, respectively (Fig. **6**). The accuracies of the areas under the ROC curve for DLBC, LAML, OV, PRAD, READ, UCS, and UVM were 0.92, 0.94, 0.93, 0.96, 0.87, 0.94, and 0.89, respectively. Although the confusion matrix showed that CHOL had only two misidentified samples in the test set, its accuracy was the lowest (0.57) for pan-cancer, indicating that accuracy not only relates to recognizing misidentified samples in the test set but also depends on the proportion of the misidentified samples in the test set. Overall, based on the neural network, the accuracies for CESCs, COAD, DLBC, GBM, KIRC, LAML, LIHC, lung adenocarcinoma, LUSC, MESO, OV, pheochromocytoma and paraganglioma, PRAD, sarcoma, SKCM, THCA, uterine corpus endometrial carcinoma, and UCS were ≥0.9. Thus, the model used to identify the outcomes of immunotherapy was improved.

## 4 DISCUSSION

This study comprehensively analyzed 59 MSC-related genes [25] in multi-omics and clinical data of multiple cancers and demonstrated the global distribution of genes controlled by MSC at the genetic, epigenetic, and transcription levels.

When exploring why MSC scores predict poor patient prognosis in multiple tumors, using multiple immune algorithms, we revealed that this may be caused by crosstalk between MSCs and tumor-associated fibroblasts. Louault *et al.* [32] found that MSCs, the precursors of cancer-associated fibroblasts, coexist with tumor-associated macrophages in untreated human neuroblastoma (NB) tumors. These tumors are also poorly infiltrated by T cells and natural killer (NK) cells. MSCs and CAF-MSCs harvested from neuroblastoma tumors protect human monocytes (MNs) from spontaneous apoptosis *via* an interleukin (IL)-6-dependent mechanism. Furthermore, MSCs and osteosarcoma cells communicate with each other through paracrine signaling mediated by cytokines, growth factors, chemokines, and EVs. This communication induces MSC migration and transformation into tumor-associated phenotypes, promotes angiogenesis and metastasis, and confers drug resistance [33]. Mei *et al.* [34] identified two MSC populations in the bone marrow of normal humans and the bone marrow of patients with clear cell renal cell carcinoma metastases, namely MSC-1 and MSC-2, respectively. MSC-1 derived from benign bone marrow expressed large amounts of CXCL12, LEPR, VCAN, SEPP1, and VCAM1. In contrast, MSC-2 clusters derived from the bone marrow of patients with clear cell renal cell carcinoma metastases maintained the classic MSC markers NT5E and THY1 (CD90), but the expression of VCAM1, LEPR, and CXCL12 was reduced. At the same time, multiple collagen-related genes (*COL6A2, COL3A1, COL4A1,* and *COL4A2*) in the MSC-2 cluster were upregulated in bone metastases, which is consistent with our observation of IGFBP3, TAGLN, LUM, COL6A1, COL6A2, and COL3A1 in pan-cancer in Fig. (**1d**). The generally high expression of COL1A1 and COL1A2 is consistent. More findings are also reflected in the fact that MSC-2 showed obvious EMT characteristic scores, especially within bone metastases. This elevated EMT score indicates a substantial degree of cell state plasticity and motility, which are considered key indicators of metastatic potential [35, 36]. After mapping MSC-2 features to bulk data, it was found that MSC-2 features were associated with poor progression-free survival and OS. In addition, Mei *et al.* [34] further pointed out that MSC-2 showed high expression of cancer-associated fibroblast markers, including FAP, FN1, and CD44, and demonstrated cancer-associated fibroblast marker expression of MSC in metastatic bone marrow. This is not observed in normal bone marrow [37].

Based on existing research and our integrated analysis of MSC pan-cancer, we tried to reveal the factors responsible for the poor prognosis of MSC in pan-cancer, hoping to provide new insights for future preclinical research and actual clinical applications.

However, the application of MSCs in clinical practice is limited by the inability to perform large-scale clinical immune-checkpoint therapy trials. Therefore, only retrospective studies of certain existing immunotherapy cohorts can be used for subsequent research. Some confounding factors that may affect immunotherapy in these cohorts remain unclear, and relevant basic research and even clinical trials on the impact of MSCs on immunotherapy need to be redesigned and evaluated in the future.

## CONCLUSION

In summary, we characterized MSCs across multiple cancer types and highlighted their potential as predictive biomarkers for immunotherapy treatment response and prognosis.

## Figures and Tables

**Fig. (2):**
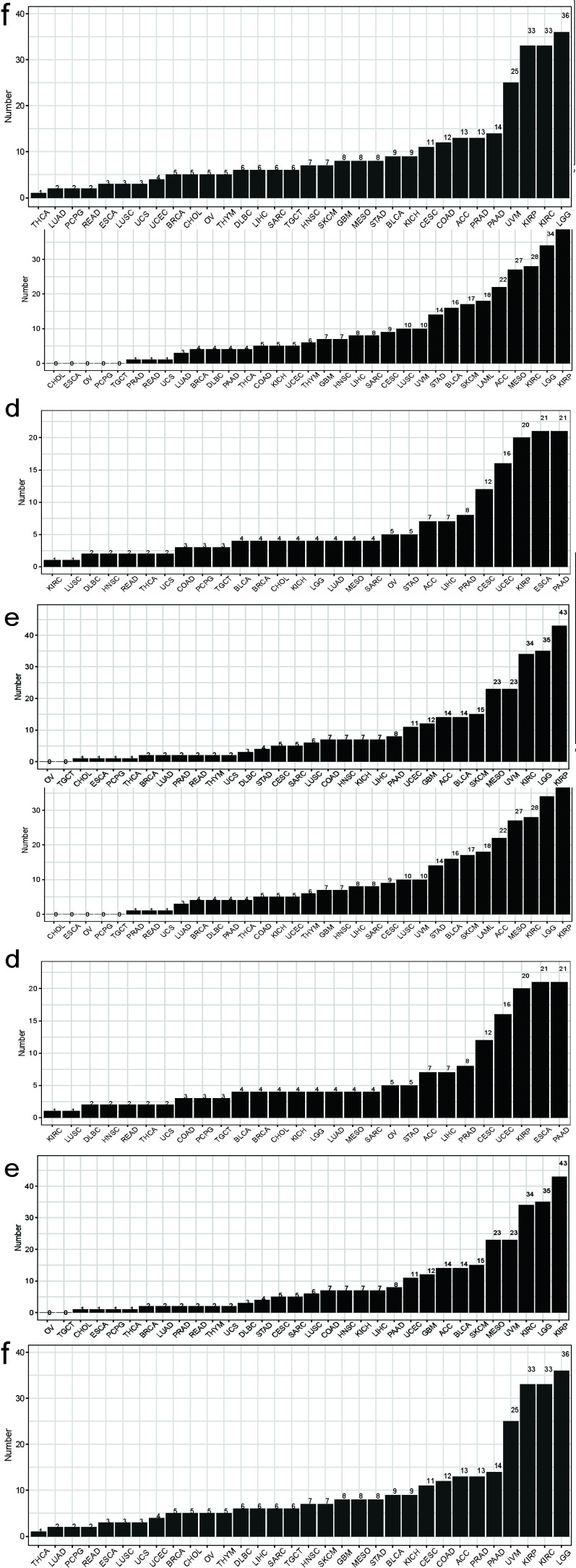
MSC-related genes with prognostic significance in pan-cancer of TCGA. (**a**) The number of prognostically significant MSC-related genes in terms of Cox regression analysis and OS, disease-free interval (DFI), DSS, and PFI analyses. Prognostically significant MSC-related genes are arranged by number for (**b**) Cox regression analysis results, (**c**) OS, (**d**) DFI, (**e**) and (**f**) PFI.

## Data Availability

The authors confirm that the data supporting the findings of this research are available within the article.

## LIST OF ABBREVIATIONS

ACC: Adrenocortical Carcinoma
BLCA: Bladder Urothelial Carcinoma
CESC: Cervical and Endocervical Cancer
CHOL: Cholangiocarcinoma
CI: Confidence Interval
CMap: Connectivity Map
COAD: Colon Adenocarcinoma
DFI: Disease-free Interval
DSS: Disease-specific Survival
GBM: Glioblastoma Multiforme
GSEA: Gene Set Enrichment Analysis
HR: Hazard Ratio
KICH: Kidney Chromophobe
KIRC: Kidney Renal Clear Cell Carcinoma
KIRP: Kidney Renal Papillary Cell Carcinoma
LIHC: Liver Hepatocellular Carcinoma
LUSC: Lung Squamous Cell Carcinoma
MESO: Mesothelioma
MSCs: Mesenchymal Stem Cells
NB: Neuroblastoma
NK: Natural Killer
OS: Overall Survival
PAAD: Pancreatic Adenocarcinoma
PFI: Progression-free Interval
PRAD: Prostatic Adenocarcinoma
READ: Rectum Adenocarcinoma
ROC: Receiver Operating Characteristic
SKCM: Skin Cutaneous Melanoma
STAD: Stomach Adenocarcinoma
TA-MSCs: Tumor-associated MSCs
TAF: Tumor-associated Fibroblast
TCGA: The Cancer Genome Atlas
TGCTs: Testicular Germ Cell Tumors
THCA: Thyroid Carcinoma
THYM: Thymoma
TME: Tumor Microenvironment
UCS: Uterine Carcinosarcoma
UVM: Uveal Melanoma
