# Supplementary material for: Integrated Analysis of Clinical Outcome of Mesenchymal Stem Cell-related Genes in Pan-cancer
Source: Curr Genomics. 2024 Apr 26;25(4):298–315. doi: 10.2174/0113892029291247240422060811 (PMC11327807; doi:10.2174/0113892029291247240422060811)
Supplement: Supplementary file 1 [file CG-25-298_SD1.zip › CG-25-298_SD1/3b-BMS-CG-2023-194 Supplementary Material Figures.pdf]

Supplementary Material

Integrated Analysis of Clinical Outcome of Mesenchymal Stem Cell-related Genes in Pan-cancer

Mingzhe Jiang[1], Dantong Zhu[1], Dong Zhao[1], Yongye Liu[1], Jia Li[1] and Zhendong Zheng[1,*]

[1]*Department of Oncology, General Hospital of Northern Theater Command, Shenyang, China*

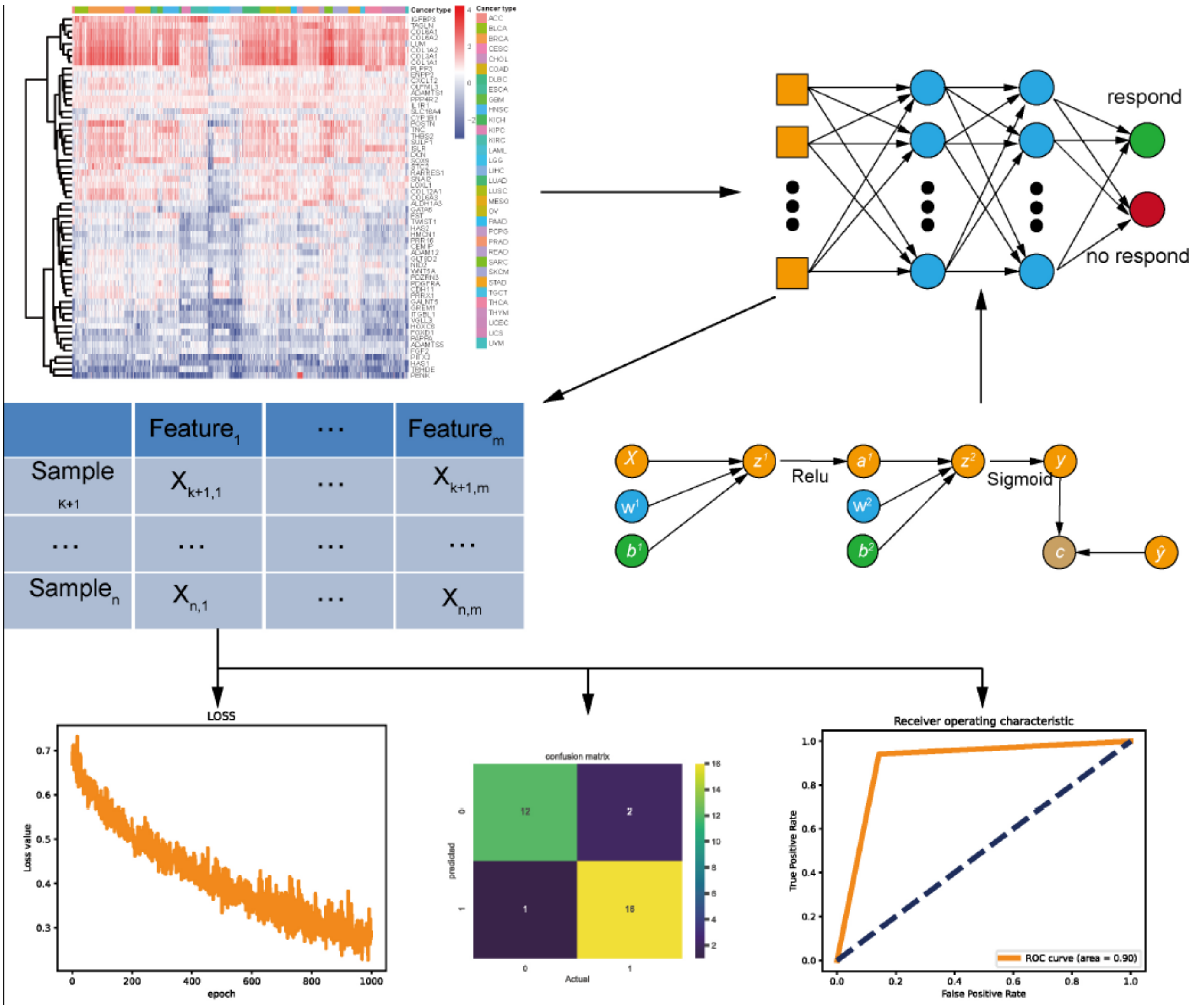

Supplementary Figure 1. Schematic diagram of neural network.

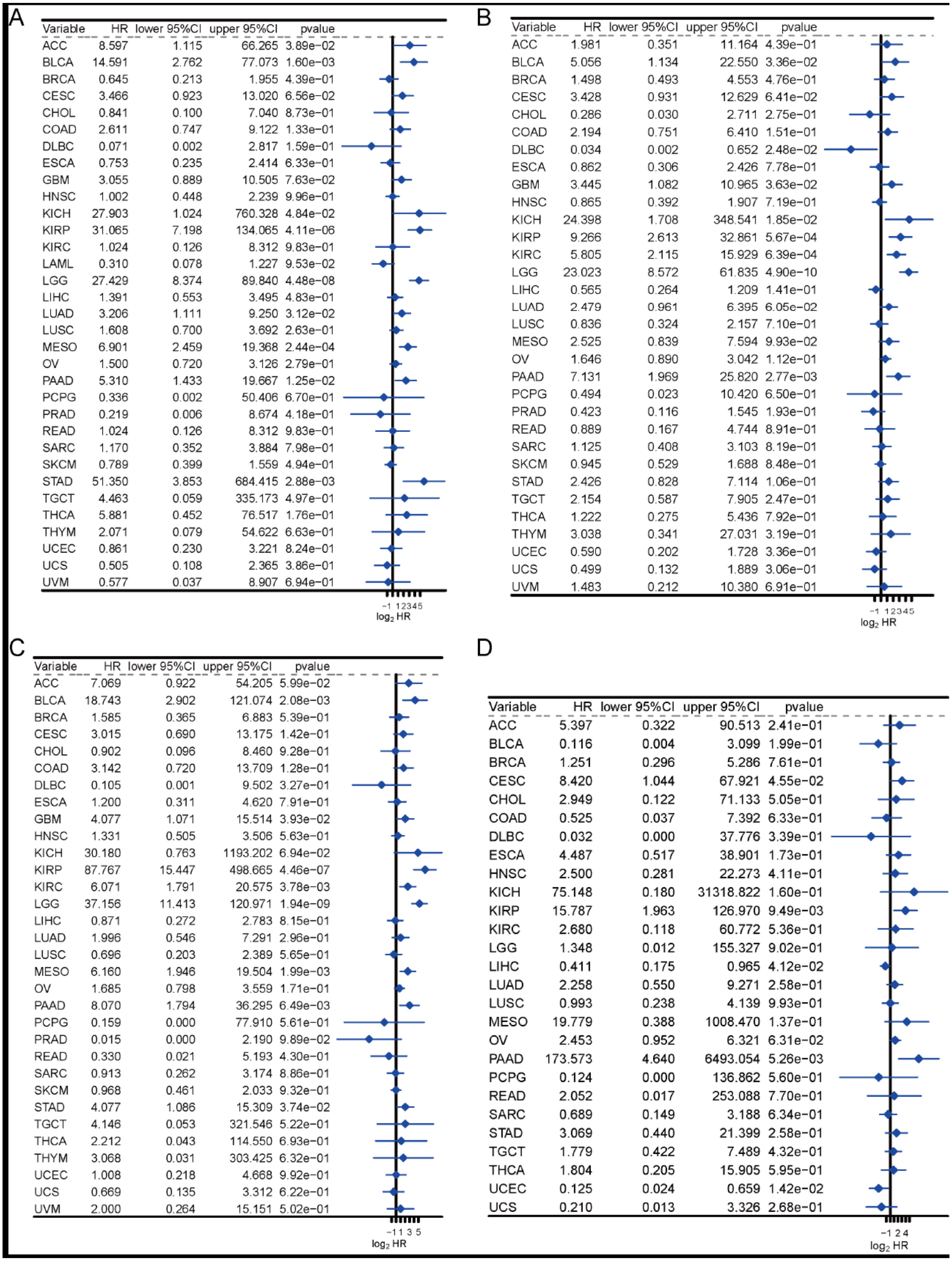

Supplementary Figure 2. Univariate Cox regression analysis of MSC scores in pan-cancer **(A)** OS, **(B)** PFS, **(C)** DSS, and **(D)** DFI for univariate Cox regression analysis in pan-cancer.

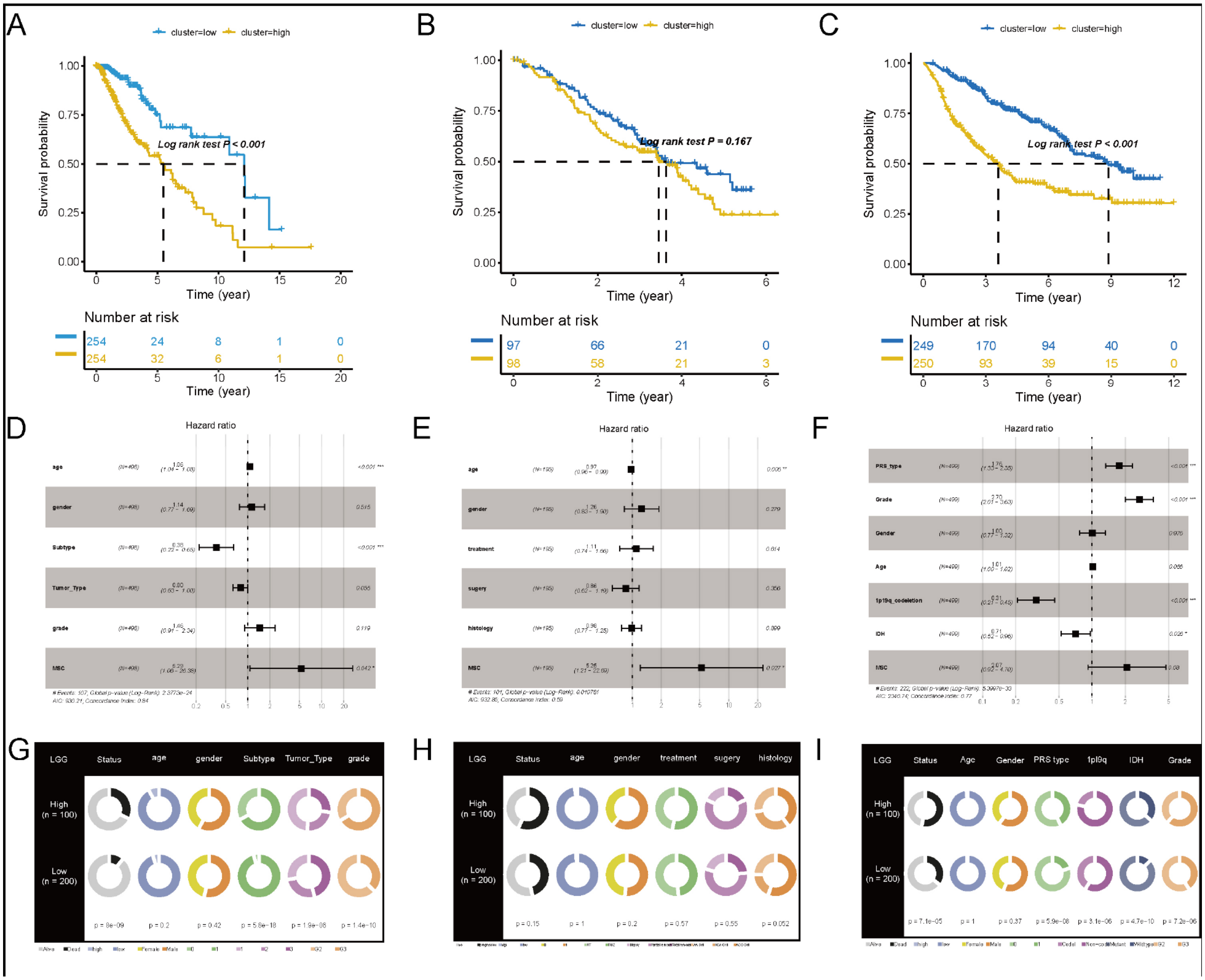

Supplementary Figure 3. Independent prognostic factor analysis for LGG Overall survival (OS) in **(A)** TCGA, **(B)** GSE107850 and **(C)** CGGA between high and low MSC score group, multivariate regression analysis for MSC score and clinical characteristics in **(D)** TCGA, **(E)** GSE107850 and **(F)** CGGA, Fisher's test between MSC score and clinical characteristics in **(G)** TCGA, **(H)** GSE107850 and **(I)** CGGA.

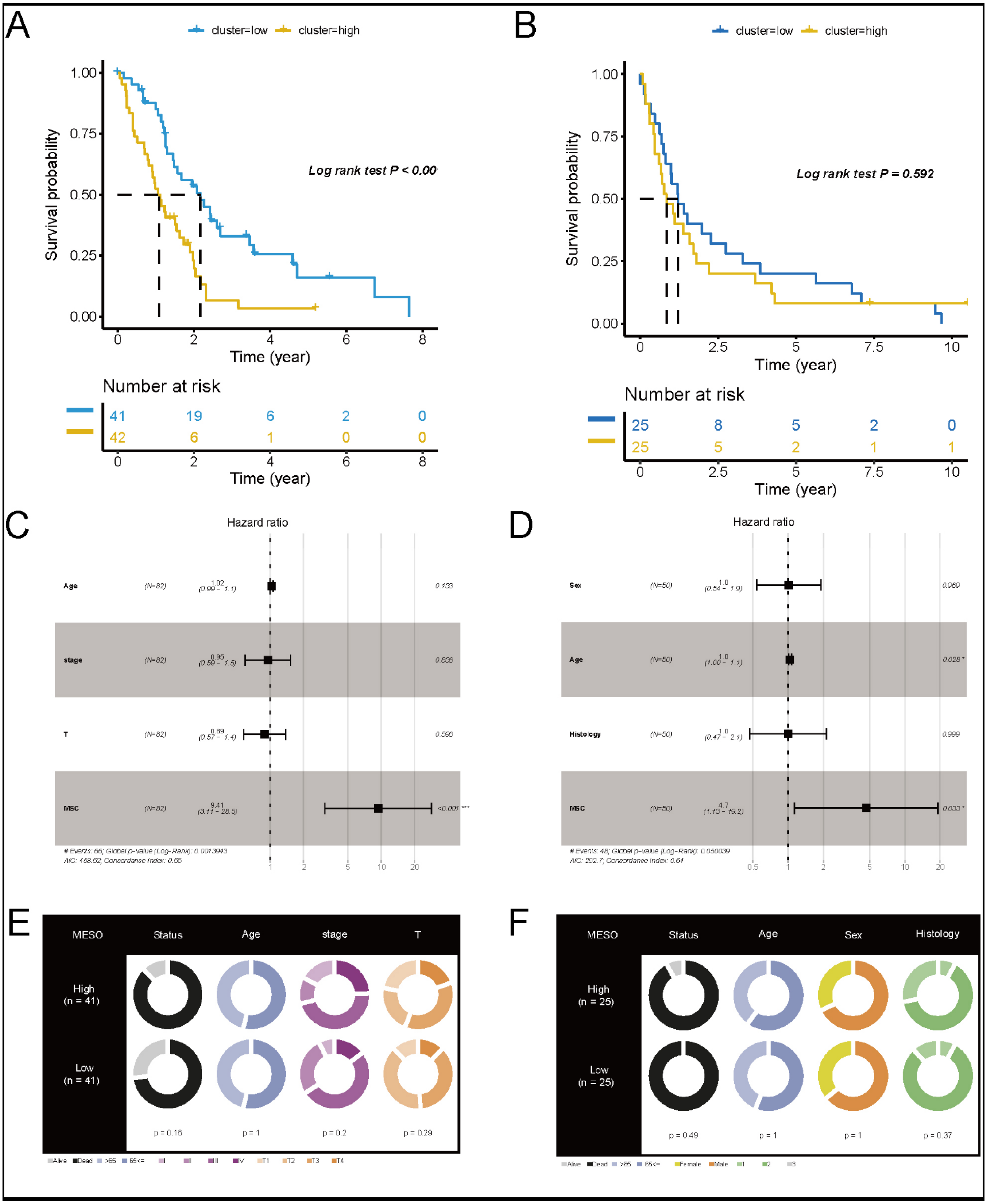

Supplementary Figure 4. Independent prognostic factor analysis for MESO Overall survival (OS) in **(A)** TCGA and (B) GSE29354 between high and low MSC score group, multivariate regression analysis for MSC score and clinical characteristics in **(C)** TCGA and **(D)** GSE29354, Fisher's test between MSC score and clinical characteristics in **(E)** TCGA and **(F)** GSE29354.

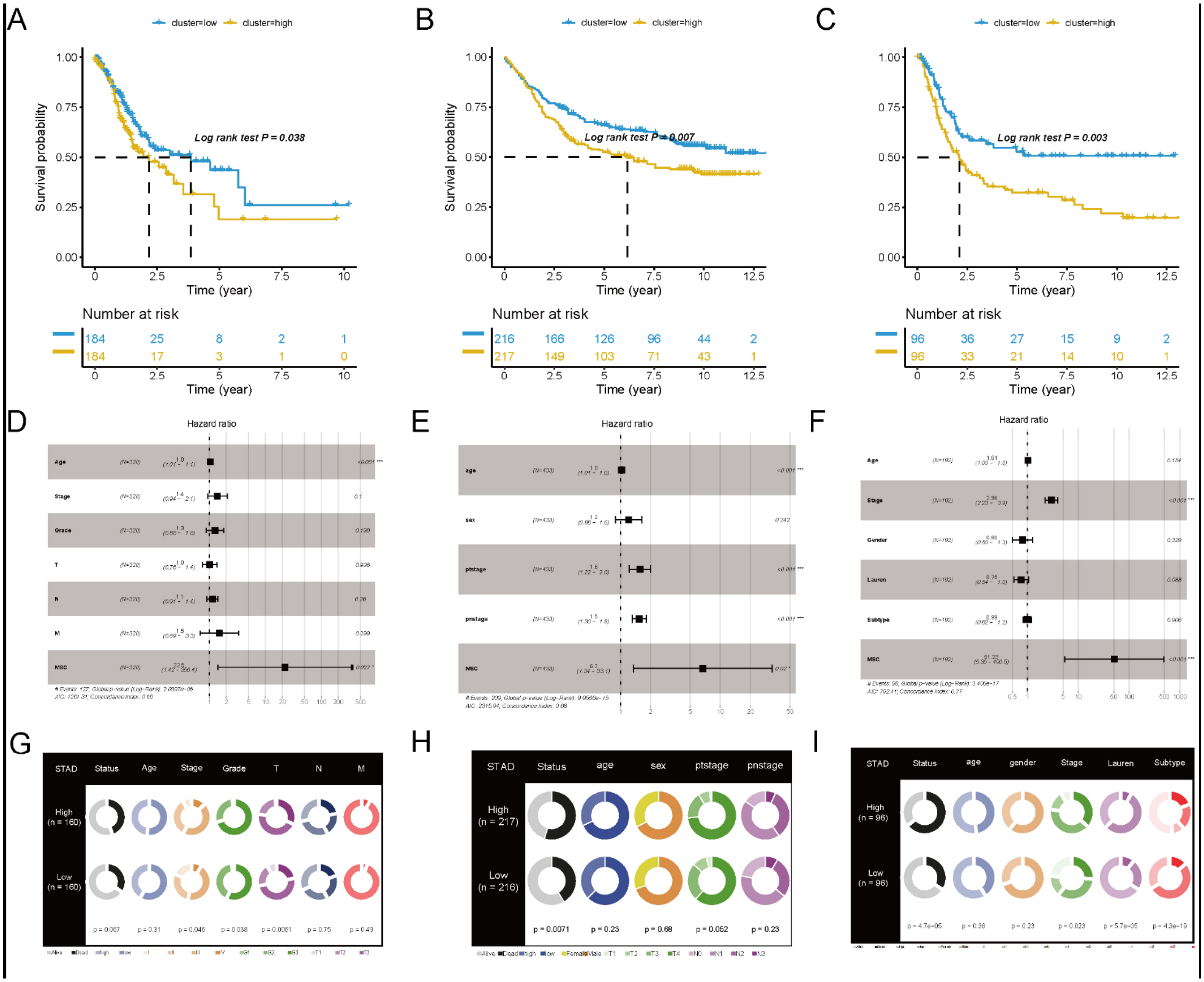

Supplementary Figure 5. Independent prognostic factor analysis for STAD. Overall survival (OS) in **(A)** TCGA, **(B)** GSE84437 and **(C)** GSE15459 between high and low MSC score group, multivariate regression analysis for MSC score and clinical characteristics in **(D)** TCGA, **(E)** GSE84437 and **(F)** GSE15459, Fisher's test between MSC score and clinical characteristics in **(G)** TCGA, **(H)** GSE84437 and **(I)** GSE15459.

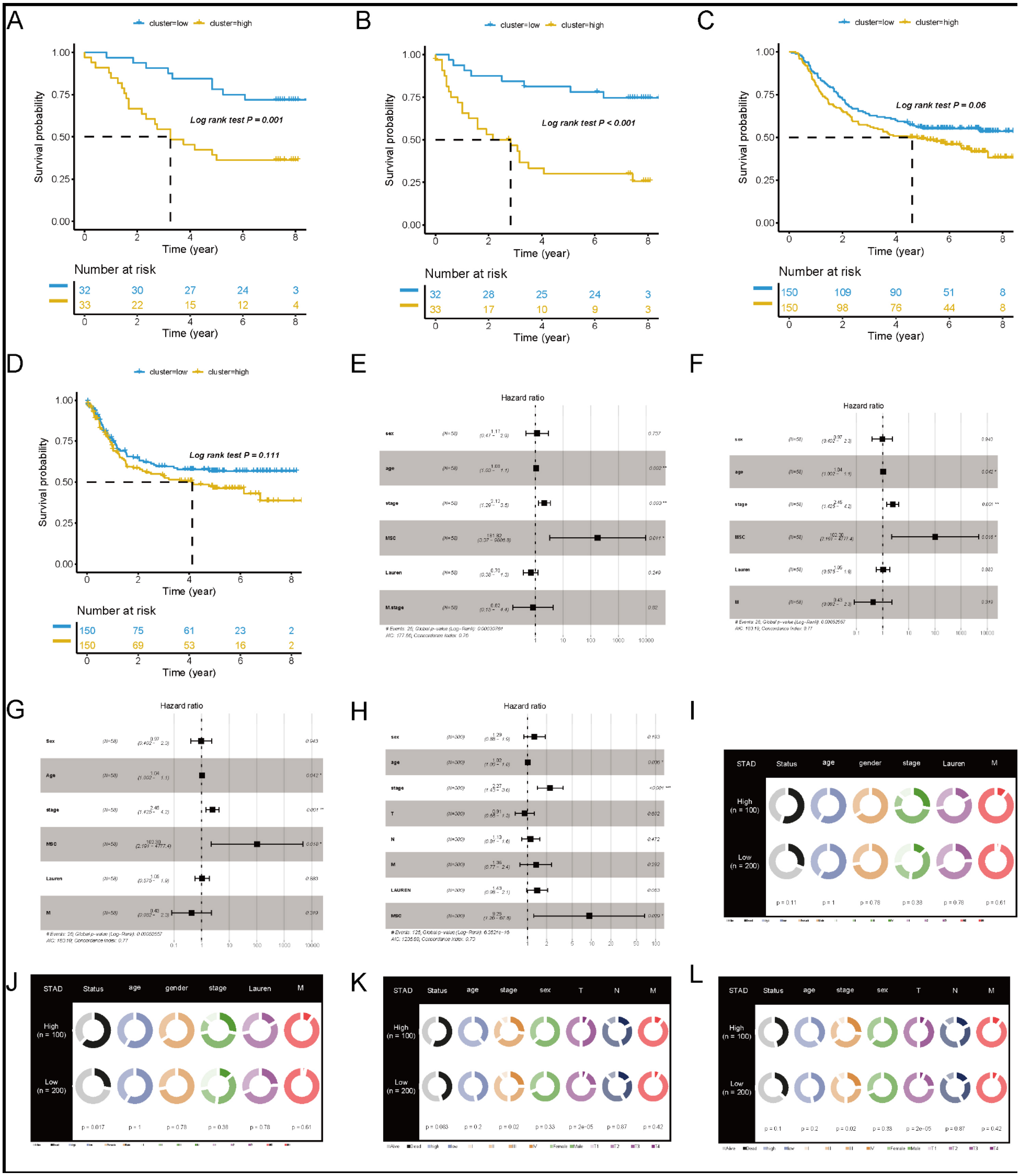

Supplementary Figure 6. Independent prognostic factor analysis for STAD. **(A)** Overall survival (OS), **(B)** relapse-free survival (RFS) in GSE13861, **(C)** OS and **(D)** RFS in GSE62254 between high and low MSC score group, multivariate regression analysis for MSC score and clinical characteristics in **(E)** GSE13861-OS, **(F)** GSE13861-RFS, **(G)** GSE62254-OS, and **(H)** GSE62254-RFS. Fisher's test between MSC score and clinical characteristics in (I) GSE13861-OS, **(J)** GSE13861-OS, **(K)** GSE62254-OS and **(L)** GSE62254-RFS.

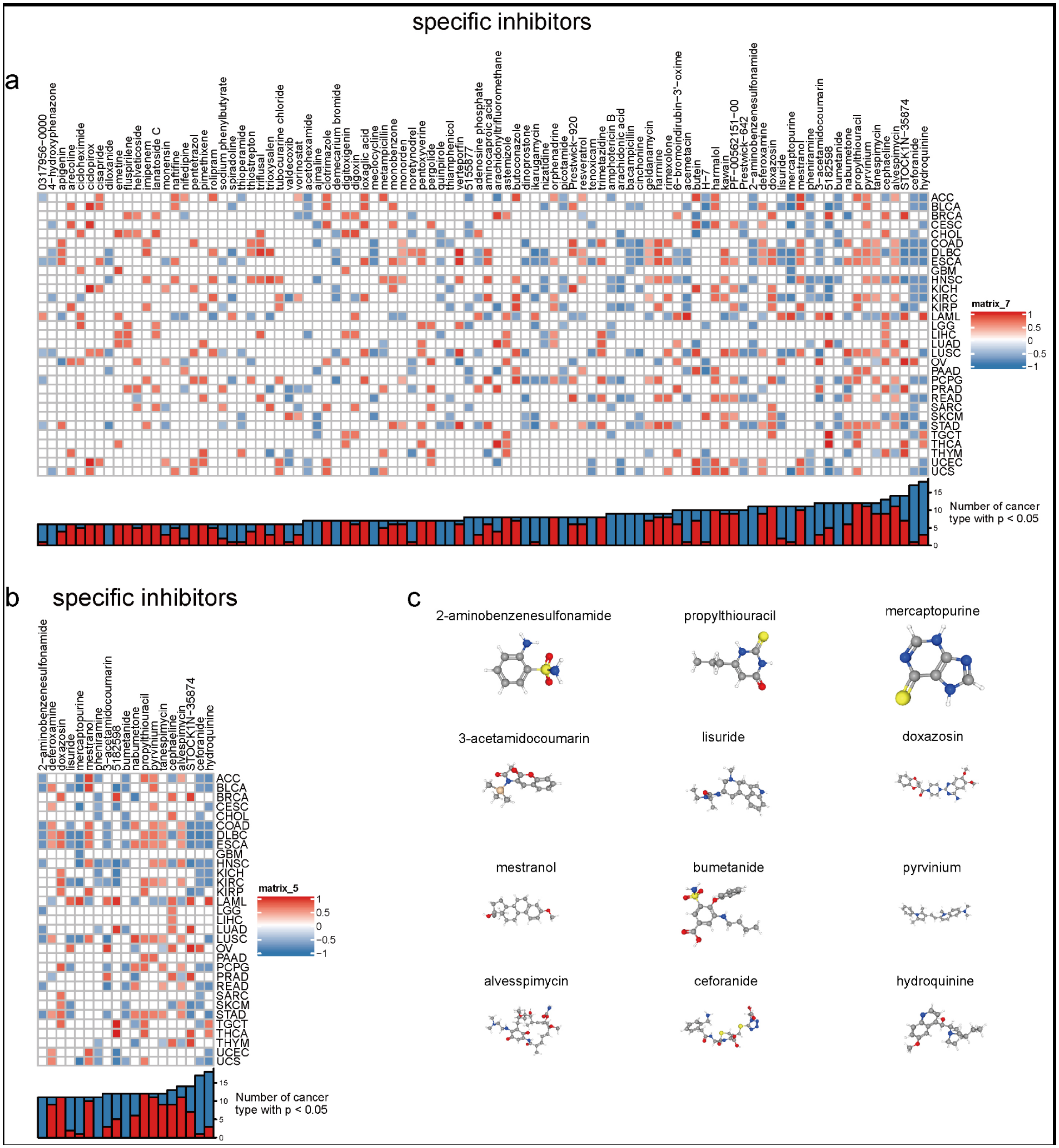

Supplementary Figure 7. CMap analysis to select novel drugs in pan-cancer Heatmap showing the enrichment score (positive, blue; negative, red) of each compound from the CMap for each cancer type. Compounds are sorted from right to left by the descending number of significantly enriched cancer types. (A) Compounds significantly enriched in more than five cancer types. (B) Compounds significantly enriched in >10 cancer types. (C) The three-dimensional structure of the 12 small-molecule drugs.

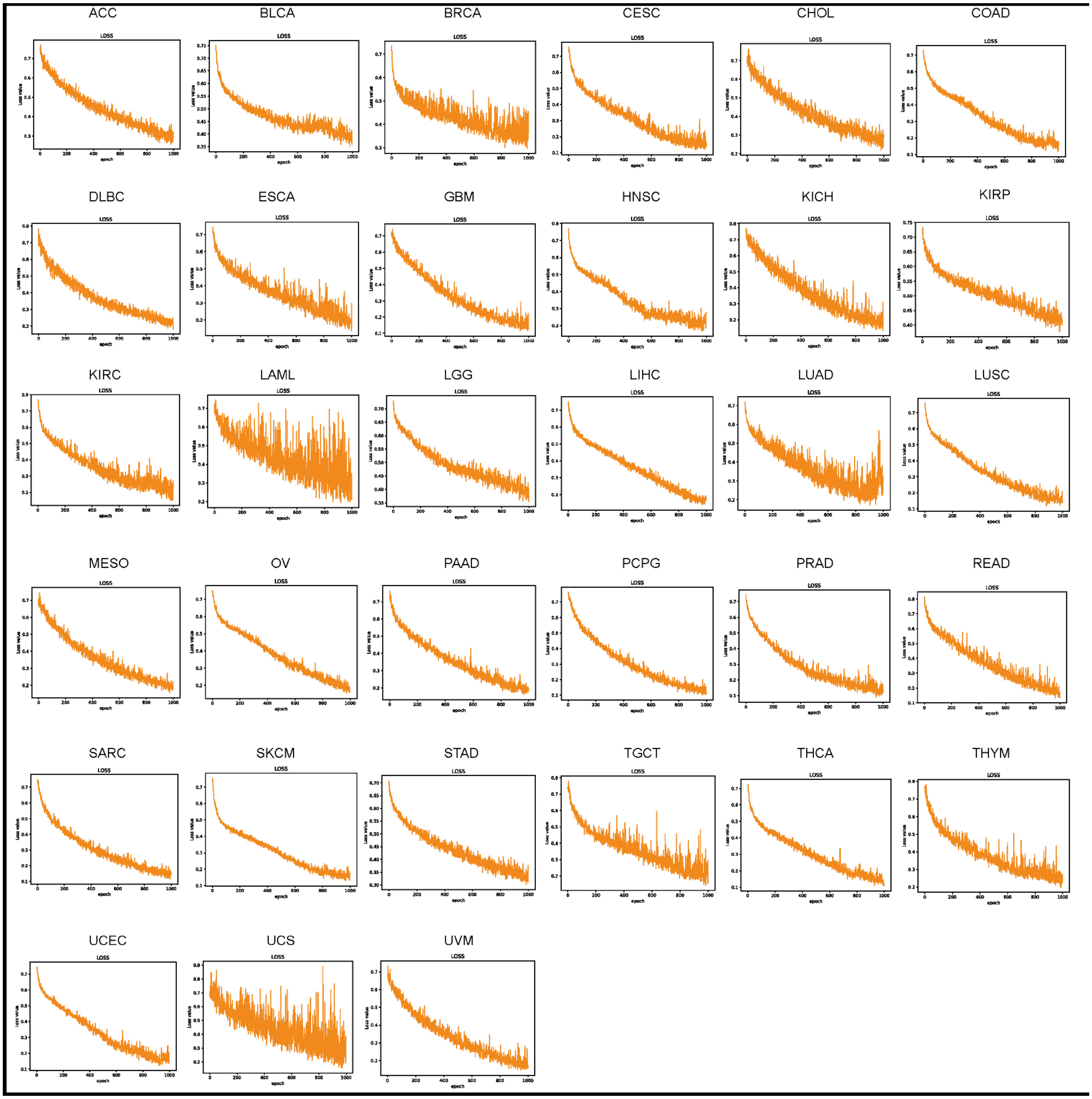

Supplementary Figure 8. The combined use of the TIDE algorithm and neural network to predict the accuracy of MSC-related genes for immunotherapy in pan-cancer The loss value in each epoch during the training process in the validation cohort in pan-cancer.

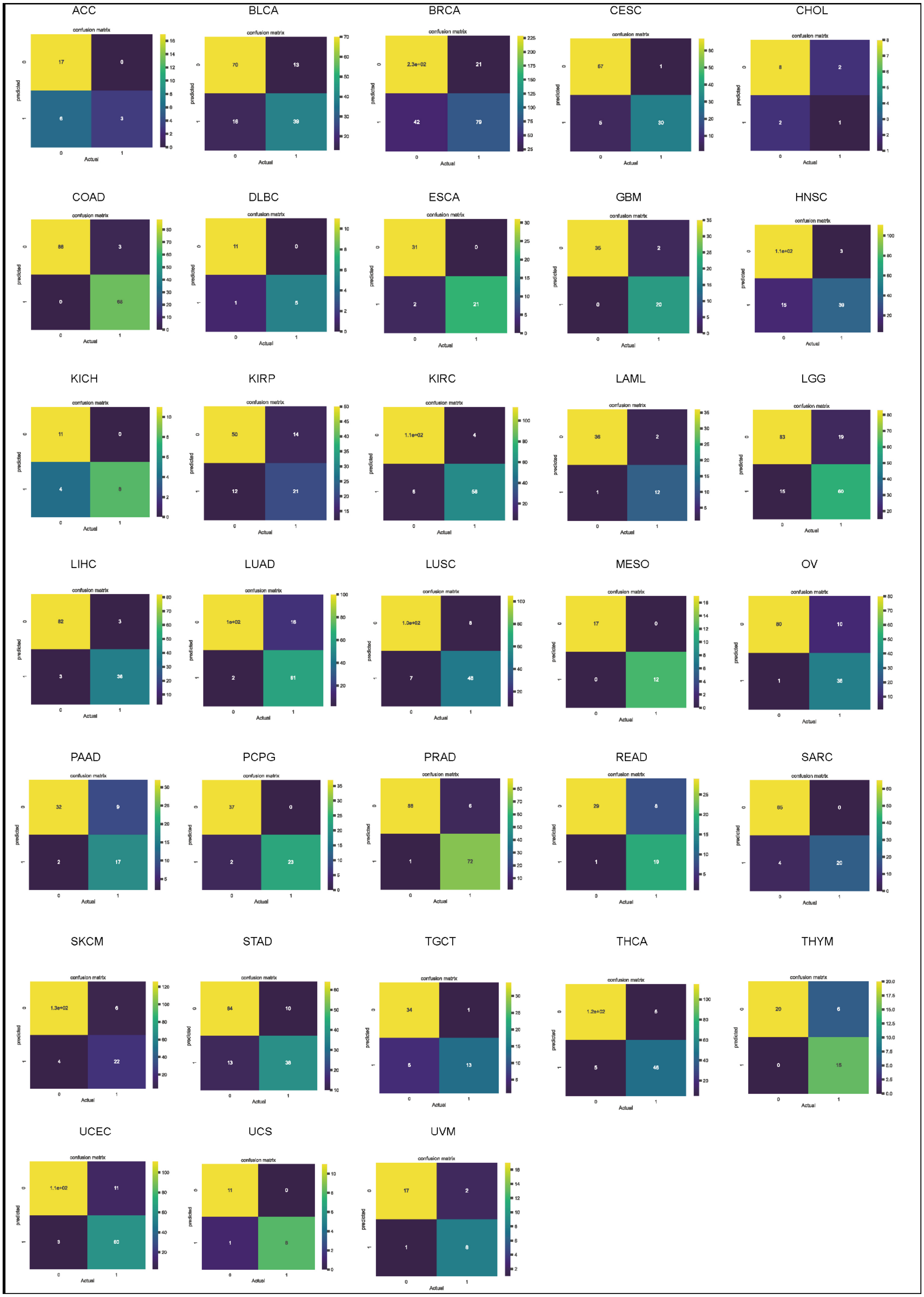

Supplementary Figure 9. The combined use of the TIDE algorithm and neural network to predict the accuracy of MSC-related genes for immunotherapy in pan-cancer. The confusion matrix in the testing cohort validated the accuracy of the network's prediction capacity in pan-cancer.
